# SARS-CoV-2 transmission dynamics in bars, restaurants, and nightclubs

**DOI:** 10.3389/fmicb.2023.1183877

**Published:** 2023-05-18

**Authors:** Brian M. J. W. van der Veer, Koen M. F. Gorgels, Casper D. J. den Heijer, Volker Hackert, Lieke B. van Alphen, Paul H. M. Savelkoul, Christian J. P. A. Hoebe, Jozef Dingemans

**Affiliations:** ^1^Department of Medical Microbiology, Infectious Diseases and Infection Prevention, Care and Public Health Research Institute (CAPHRI), Faculty of Health, Medicine and Life Sciences, Maastricht University Medical Centre (MUMC+), Maastricht, Netherlands; ^2^Department of Sexual Health, Infectious Diseases and Environmental Health, South Limburg Public Health Service, Heerlen, Netherlands; ^3^Department of Social Medicine, Care and Public Health Research Institute (CAPHRI), Faculty of Health, Medicine and Life Sciences, Maastricht University, Maastricht, Netherlands

**Keywords:** SARS-CoV-2, whole-genome sequencing, transmission dynamics, dancing facilities, bar and restaurant industry

## Abstract

**Background:**

In an attempt to control the spread of SARS-CoV-2, many governments decided to close public venues including bars, restaurants, and nightclubs during the pandemic, making it difficult to study how transmission occurs in these environments. In this study, we were able to gain insight into the transmission dynamics of SARS-CoV-2 in 16 venues in the city of Maastricht using a combination of epidemiological and whole-genome sequencing (WGS) data during a period of 2 weeks in 2021, when bars, restaurants, and nightclubs were temporarily reopened in the Netherlands. This led to a subsequent rise of SARS-CoV-2 cases in the community following the reopening.

**Methods:**

WGS was performed on samples from 154/348 of selected cases and combined with epidemiological investigation (e.g., contact tracing and linking cases to specific venues) to identify SARS-CoV-2 transmission clusters. In addition, genomic surveillance data were used to investigate spillover of outbreak-associated genotypes into the community.

**Results:**

Clustering was observed in 129/136 (95%) successfully genotyped samples. We established that most cases were linked to venues with dancing facilities and that specific genotypes of the Delta variant were more frequently spread within and from these venues compared to venues without dancing facilities. In addition, we show indications of spillover of certain genotypes from the bar and restaurant industry into the community, with the number of hospital admissions increasing in the weeks following peak cases in the community.

**Conclusion:**

Lifting restrictions on bar and restaurant industry venues with a corona entree ticket in a largely unvaccinated population led to a surge in COVID-19 cases and promoted the spread of new (sub)variants. Nightclubs were identified as potential super-spreading locations.

## Introduction

On June 26, 2021, Dutch authorities lifted social distancing and customer capacity restrictions for venues like bars, restaurants and cafes, and ending a 15 month curb on nightclubs, large gatherings, and festivals. Using a digital COVID-19 certificate, customers had to provide evidence they had completed a full vaccination scheme against SARS-CoV-2, had recovered from coronavirus disease 2019 (COVID-19) within 180 days prior to the event, or had tested negative for SARS-CoV-2 within 36 h prior to entry. Despite experts advising caution, the government decided that directly after completing a vaccination scheme, individuals were given access to these venues. To increase vaccine coverage among younger age groups, a campaign was launched called “Dansen met Janssen” (dancing with Janssen) that promoted vaccination with the Janssen vaccine (Jcovden) of which only a single dose was required. After receiving only one dose individuals had immediate access to nightlife, while actually the immune response takes at least 2 weeks. Remarkably, the number of cases reported in the Netherlands rose nearly twenty-fold from 552 cases on the 26th of June to 10,283 cases on the 10th of July. Restrictions on bars and restaurants were reinstated, while nightlife venues remained closed until the end of September.

Gatherings giving rise to large numbers of secondary infections are called super spreading events (SSE), considered to be important drivers of the COVID-19 pandemic (Althouse et al., 2020). Much remains unknown about factors that turn gatherings into SSEs, but crowded indoor events with poor ventilation seem to play an important role (Chau et al., 2021; Lendacki et al., 2021; Li et al., 2021; Feathers et al., 2022; Ou et al., 2022). Nightclubs in particular appear to be prone to SSEs, as shown by a number of published outbreak investigations (Kang et al., 2020; Muller et al., 2020; Nsekuye et al., 2021; Koopsen et al., 2022). To control SSEs, countries closed nightclubs while opening bars, restaurants, and large (outdoor) gatherings if physical distance between visitors could be maintained. Early nightclub outbreaks were associated with the original (wild-type) SARS-CoV-2 variant. Variant of concern (VOC) B.1.617.2, now commonly referred to as the Delta variant, rapidly increased its foothold during our study period. With viral loads exceeding those of the original (wild-type) Wuhan strain by a factor of four (von Wintersdorff et al., 2022), and almost twice its transmissibility (Campbell et al., 2021), Delta appears to greatly facilitate SSEs. Moreover, individuals infected with the Delta variant are at increased risk of hospitalization (Twohig et al., 2022), putting health-care services under additional pressure. Even in a largely vaccinated population, Delta may be associated with clusters during large gatherings (Siddle et al., 2022). Therefore, gaining insights into the transmission dynamics of large events is very important.

In this paper, we describe outbreaks associated with the Delta variant in a largely unvaccinated population, occurring in night clubs, bars and restaurants in the Dutch city of Maastricht, in the period from June 26, 2021 to July 10 when restrictions were reinstated. To elucidate transmission dynamics in these outbreaks, we combined epidemiological analysis with whole-genome sequencing (WGS) data.

## Materials and methods

### Setting

With approximately 120.000 inhabitants, Maastricht is the largest city in South Limburg, the southernmost region of the Netherlands. The city, which is home of a large university, has an active student population of more than 21.000 students, and a busy nightlife. Full vaccination coverage against COVID-19 among individuals younger than 30 years was below 20% at the time of the study (June 26, 2021 to July 10, 2021; based on our own data), while approximately 50–60% had started a vaccination scheme. Vaccination is freely available for individuals younger than 30 years since June 2021. The regional Public Health Service (PHS) provided a range of free-of-charge services, such as COVID-19 testing and vaccination against COVID-19 for all individuals aged 12 years and above. In addition, the PHS performed source and contact tracing for all notified cases of COVID-19, and initiated, supervised or provided consultation on regional control efforts, including the closure of public venues.

### Epidemiological investigation

Cases were defined as individuals that visited at least one catering venue between June 26 and July 10, and were tested positive for SARS-CoV-2 (between June 26 and July 24 by RT-PCR or antigen test). Catering venues were categorized into nightclubs, restaurants/bars, combination venues, or student association venues. No physical distancing rules applied to nightclubs but visitors had to show a digital COVID-19 certificate to gain access. In contrast, customers were obliged to maintain 1.5 m distance in restaurants/bars but no COVID-19 certificate was required. Venues entertaining mixed day- and nighttime activities were classified combination venues. Student association venues provided a mixture of activities, including nighttime entertainment or parties requiring a digital COVID-19 certificate for entry. During source and contact tracing all notified cases were asked which venues they attended in the 7 days prior to symptom onset or positive test and were subsequently linked to that venue. They were also asked what they considered to be the most likely source of their infection. Venues with at least 10 linked cases were included in this study.

Based on Dutch guidelines, the contagious and incubation period were defined as 2 days and 2–14 days prior symptoms, respectively. COVID-19-related symptoms were defined as cough, common cold symptoms, fever (>38°C), loss of taste or smell, diarrhea, nausea, fatigue, and headache. Each case was questioned regarding kind and severity of symptoms, symptom onset, COVID-19 vaccination status, immune status, and the kind and number of venues visited during their incubation and contagious periods. Cases were further categorized into staff members of venues and visitors. Staff members were defined as any employee working in at least one of the venues they visited.

Per case, the total amount of bar and restaurant industry venue visitations was calculated, including venues not in this study. If a case visited a venue on multiple days each visit was counted. The visits were further stratified based on occurrence during source or contagious period but contact tracers only collected venue data 7 days prior to symptom onset.

We considered cases as fully vaccinated if they had received their second dose of Comirnaty (Pfizer), Spikevax (Moderna), or Vaxzevria (Astrazeneca) or a single dose of Jcovden (Janssen) at least 14 days before symptom onset. Individuals who had recovered from COVID-19 in the 6 months prior to vaccination only required one dose to be considered fully vaccinated. Partially vaccinated were cases who had received one vaccination of Comirnaty, Spikevax, or Vaxzevria. Reinfection was defined as two positive tests at least 8 weeks apart.

Digital COVID-19 certificates were granted to individuals who had recovered from COVID-19 in the past 180 days, tested negative within 36 h, or completed a full vaccination scheme. No incubation period after vaccination was required to receive this certificate. With this certificate, individuals could attend events where physical distancing was not required, but venue staff were exempt to show the certificate. An analysis was performed on cases that had visited a venue during their contagious period and preceding symptoms to test effectiveness of the certificate.

### Next-generation sequencing of SARS-CoV-2-positive samples

All available samples from the two largest analysis facilities (MUMC+ and Synlab) were included for sequencing apart from antigen tests or samples with a cycle threshold value > 32. Sequencing was performed as previously described (von Wintersdorff et al., 2022) using the PCR tiling of SARS-CoV-2 virus with Native Barcoding Expansion 96 (EXP-NBD196) protocol (Version: PTCN_9103_v109_revH_13Jul2020) of Oxford Nanopore technologies, with minor modifications and using the primers published by Oude Munnink et al. (2020).

The quality of the SARS-CoV-2 WGS protocol described here was evaluated *via* participation in an external quality assessment across 15 European laboratories and found to be 100% accurate for cluster identification (Wegner et al., 2022).

SARS-CoV-2 isolates were considered to be part of the same cluster/genotype if there was ≤2 SNPs difference. However, epidemiological links were also taken into account to classify sub-clusters of genotypes with 1 or 2 SNPs difference.

### Regional genomic surveillance of SARS-CoV-2 variants

Genomic surveillance of SARS-CoV-2 variants in the South-Limburg region was performed on a weekly basis continuously throughout 2021. This was achieved by randomly selecting positive samples from the community with a sufficiently high viral load (CT-value <30). To ensure good regional coverage, about 10% of positive samples were sequenced.

### Determination of viral load

Viral load was determined *via* RT-PCR analysis as previously described (Dingemans et al., 2022). Only samples that were tested using the same laboratory workflow were included to compare the viral load in this study.

### Statistical analysis

A Kruskal–Wallis test was performed to compare the median cycle threshold (Ct) values of the N-target of fully vaccinated, partially vaccinated and non-vaccinated cases. To account for different testing methods in multiple labs only samples were included from one lab which tested the great majority of our samples (276/348, 79%). *p*-values <0.05 were considered significant.

### Ethical statement

All data presented in this paper, including information obtained from affected institutions, were retrospectively retrieved from regular infectious disease control activities and were de-identified. As such, our study does not fall under the scope of the Dutch Medical Research Involving Human Subjects Act (WMO) and therefore is exempt from medical ethical approval. This was confirmed by the Maastricht University Medical Centre Medical Ethics Committee (METC 2021–2901).

## Results

### Epidemiological investigation

Sixteen venues were included, with numbers of linked cases ranging between 10 and 98 (Table 1). A total of 348 unique cases (313 visitors, 35 staff members) were linked to these 16 venues. For the entire region of South Limburg, a total of 468 cases were identified to have visited a catering venue in the study period, resulting in a coverage of 74% (348/468) for cases limited to the city of Maastricht in venues with 10 or more cases.

**Table 1:** Overview of venues included in this study.

Venue	Type of location	Visitor cases^a^	Staff member cases	Temporarily closed^b^
1	Nightclub	43	3	Yes, July 4
2	Nightclub	62	1	No
3	Nightclub	96	2	No
4	Nightclub	39	5	Yes, July 7
5	Nightclub/restaurant	57	4	No
6	Nightclub/restaurant	29	3	Yes, July 5
7	Nightclub/restaurant	50	0	No
8	Nightclub/restaurant	34	8	No
9	Student club house	11	0	No
10	Student club house	16	2	No
11	Student club house	14	1	No
12	Restaurant/Bar	10	0	No
13	Restaurant/Bar	13	1	No
14	Restaurant/Bar	13	2	No
15	Restaurant/Bar	25	2	No
16	Restaurant/Bar	13	1	No

a Visitor cases are not unique as a number of them visited multiple venues.

b Some venues were temporarily closed after the occurrence of multiple cases at their location.

Baseline characteristics of cases are displayed in Table 2. Our sample comprised 313 visitors and 35 staff members of these venues. Around 18% (55/313) of visitors were fully vaccinated >2 weeks prior to infection and 17% (53/313) of visitors had received their final vaccine dose in the 2 weeks prior to infection, of whom 89% (47/53) was vaccinated with the Janssen vaccine (Jcovden).

**Table 2:** Baseline characteristics of cases stratified by visitors and staff members.

	Visitors (*n* = 313)	Staff members (*n* = 35)
Male	161 (51%)	21 (60%)
Mean age	22.1 (range 16–58)	23.4 (range 15–53)
*Vaccination status*		
*Fully vaccinated (>2 weeks after completing a vaccination scheme)*	55 (18%)	6 (17%)
Vaxzevria	19	0
Comirnaty / Spikevax	18	2
Jcovden	17	4
*Partially vaccinated (<2 weeks after completing a vaccination scheme)**^a^*	53 (17%)	5 (14%)
Vaxzevria	1	1
Comirnaty / Spikevax	5	0
Jcovden	47	4
*One vaccination*	124 (40%)	14 (40%)
Vaxzevria	2	1
Comirnaty / Spikevax	122	13
*No vaccination*	81 (26%)	10 (29%)
*Other characteristics*		
Reinfection	8 (3%)	1 (3%)
Symptomatic	289 (92%)	34 (97%)
Asymptomatic	24 (8%)	1 (3%)
Asymptomatic and fully vaccinated	9/61 (15%)	0
Asymptomatic and not fully vaccinated	15/277 (5%)	1
*Venues visited*		
Total bar and restaurant industry venues visited during study period (median)^b^	2 (range 1–11)	3 (range 1–8)
Bar and restaurant industry venues visited during source period (median)^b^	1 (range 0–8)	2 (range 0–8)
Bar and restaurant industry venues visited during contagious period (median)^b^	0 (range 0–10)	1 (range 0–6)

a This group was eligible for a digital corona entree ticket.

b 33 visitor cases and 2 staff member cases were excluded due to incomplete information on the dates they visited the bars.

Fully vaccinated individuals (>2 weeks prior to infection) had a 15% (9/61) chance of being asymptomatic versus 6% (16/277) of the non-fully vaccinated. No cases were hospitalized during the study period. The median amount of venues visited was 2 but the range was large as some cases visited a venue more than 10 times during their contagious period. A similar spread was seen in the number of venues visited during the contagious period with one case visiting 10 venues during the contagious period. 35% (*n* = 109/313) of visitors and 60% (*n* = 21/35) of staff members visited at least one venue during their contagious period. 31% (*n* = 98/313) of visitor cases visited multiple venues on the same night. In total 21/35 (60%) staff members worked during their contagious period.

An additional analysis was conducted on cases who visited at least one venue during their contagious period. This group comprised 109 visitors and 21 staff members. 19/109 (17%) visitors were fully vaccinated versus 3/21 (14%) staff members (Table 3). The proportion of cases that was eligible for a corona entree ticket <2 weeks after vaccination was lower than in the overall study population.

**Table 3:** Overview of the status of cases visiting at least one venue during their contagious period.

Cases that visited at least one venue during contagious period^a^	Visitors (*n* = 109)	Staff members (*n* = 21)
Fully vaccinated and eligible for corona entree ticket (>2 weeks after completing a vaccination scheme)	19 (17%)	3 (14%)
Not fully vaccinated and eligible for corona entree ticket (<2 weeks after completing a vaccination scheme)	13 (12%)	1 (5%)
Not eligible for a corona entree ticket (one vaccination^*^ or no vaccination)	77 (71%)	17 (81%)

a The contagious period was defined as the 2 days prior to onset of symptoms until the end of isolation. ^*^excluding Janssen vaccine.

### Role of venues in SARS-CoV-2 transmission

Four venues were nightclubs, four were a nightclub/restaurant combination, three were student club houses, and five were bars/restaurants (Table 1). A majority of included venues (11/16, 69%) used the digital COVID-19 certificate for access. Three venues were temporarily closed after outbreaks were established at their location. The average absolute number of linked cases was higher in nightclub or nightclub/restaurant combinations.

### Using whole-genome sequencing to reveal transmission clusters

**Figure 1:**
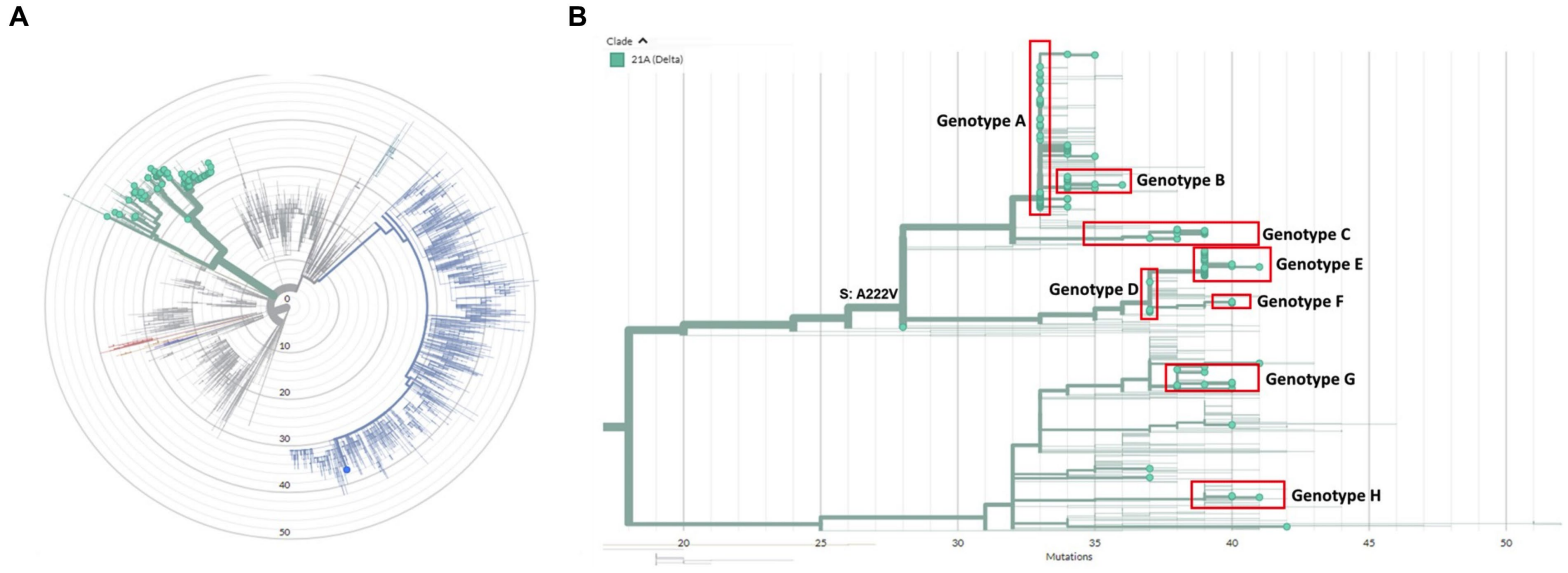
**(A)** Radial phylogenetic tree of all sequenced isolates in this study. All isolates belonged to the Delta variant (green) except for one case who was co-infected with a great majority of the Alpha variant (blue). **(B)** Detailed phylogenetic relationship of all Delta isolates in this study. Eight clusters could be identified, which were designated genotype A-H and are highlighted in red. The phylogenetic tree was visualized using the auspice tool from Nextstrain (https://auspice.us/).

**Figure 2:**
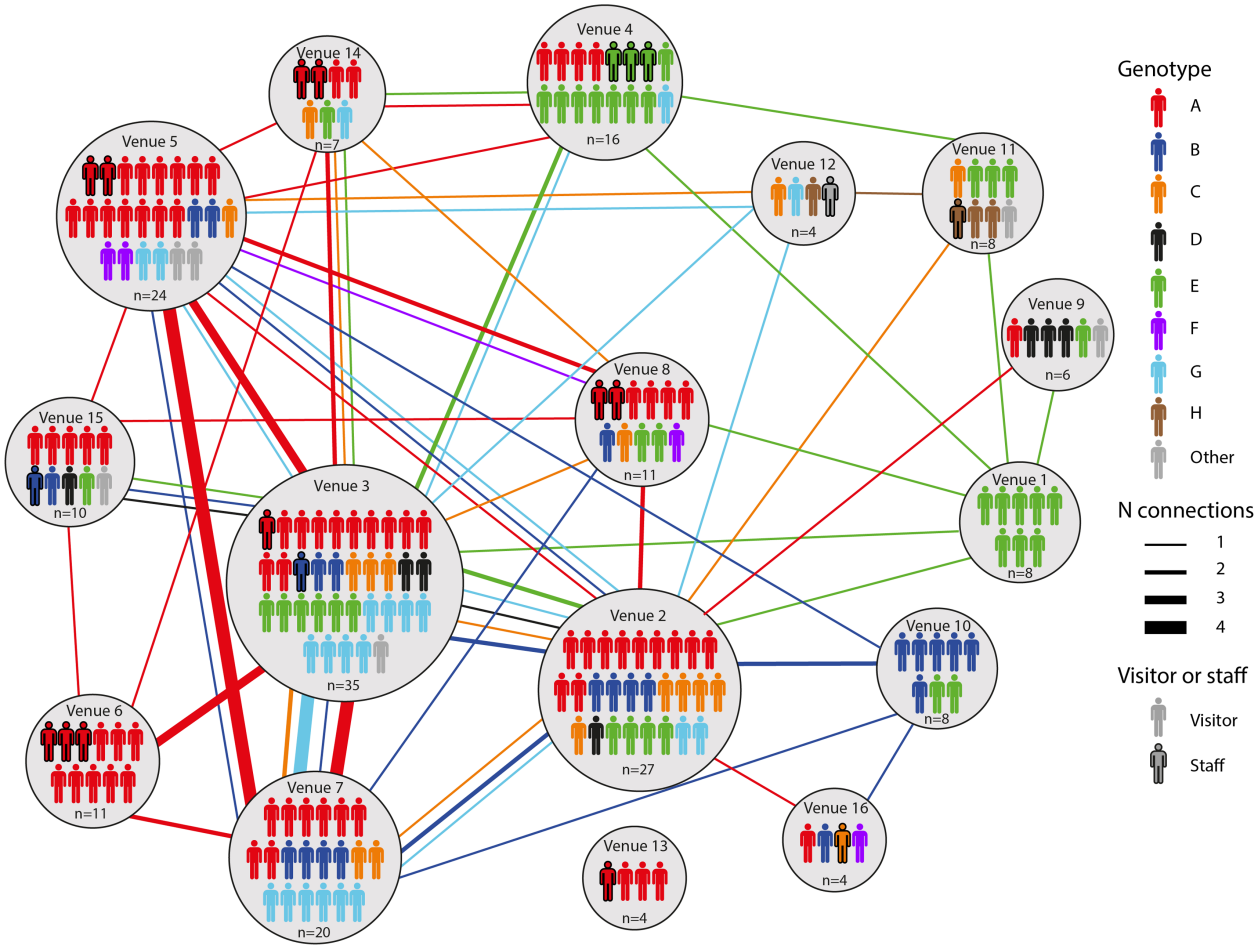
An overview of all genotypes identified in this study and the venues they were associated with. Lines between venues indicate that a particular case visited both venues.

A total of 154 cases were selected for WGS, using one sample per case. We observed no difference in age or numbers of venues when comparing cases selected for WGS and cases that were not selected (data not shown). WGS was successfully performed on 136/154 (88%) samples. All samples harbored the B.1.617.2 (Delta) variant except for one case who was co-infected with B.1.1.7 (Alpha variant) (Figure 1). Clustering, defined as two or more SARS-CoV-2 isolates of the same genotype, was observed in 129/136 (95%) samples. Genotype B was a sub-cluster of genotype A (1 SNP difference) that was characterized by amino acid substitution L1265I in the Spike protein and genotype E which was a sub-cluster of genotype D (2 SNPs difference) based on the size of these sub-clusters and the epidemiological links between cases harboring these genotypes (Figure 1). A total of eight genotypes (referred to as A to H) were thus identified, ranging in size from three (genotype F and H) to 55 cases (genotype A). Venues and associated clusters are summarized in Figure 2 (a visualization of Figure 2 is also available in Movie S1).

All genotypes were associated with multiple venues, ranging from two to 12 venues (Supplementary Figures S1–S8). Many cases visited multiple venues. Genotype A was the most representative genotype as it was identified in 55/136 (40%) cases and spread among 12/16 venues (Supplementary Figure S1). Furthermore, this genotype was found in 11/11 (100%) cases in venue 6 and in 15/24 (63%) of the cases in venue 5 as shown in Supplementary Figure S1. Genotype E, comprising 28 samples clustered most prominently in venues 1 and 4, comprising all and most of the reported cases in these venues, respectively (Supplementary Figure S5).

### Spatio-temporal transmission dynamics in bars, restaurants, and nightclubs

By combining epidemiological and WGS data, we show insight into the transmission dynamics over time in bars, restaurants, and nightclubs. Movie S1 shows a time-lapse of the movement of each successfully genotyped case between venues. On the night from the 25^th^ to the 26^th^ of June, when the bar and restaurant industry reopened, an employee of venue 14 who was later confirmed to be infected with genotype A visited venue 3. Three additional cases with genotype A visited venue 3, venue 4, and venues 2 and 8, respectively, while a first case with genotype E visited venues 3 and 4 on the same night and venue 14 one night later. Four individuals with genotype A, including the aforementioned employee of venue 14, visited venue 6 on the 29th of June. By the 1st of July, genotype E was spread among nine different venues, with cases visiting multiple places serving as links between these venues. Cases infected with genotypes C, F, G and H emerged on the 2nd of July, while cases with genotype B and D were first detected in venues on the 4th and 5th of July, respectively. Venues 2 and 3, both nightclubs, showed most genotypic diversity (6 different genotypes) among cases (Figure 2). In addition, these venues had the most cases being linked to at least another venue in which the same genotype was identified on the same night (18 and 29 respectively), indicating they acted as transmission hubs (Supplementary Table S1). In general, the number of linked cases with a particular genotype was higher for venues with dancing facilities (4–29 linked cases for venues 1–8) compared to those without (0–6 linked cases for venues 9–16). Also the number of other venues linked through genotyped cases was higher for venues with vs. those without dancing facilities (4–10 versus 0–5 linked venues, respectively).

### Genomic surveillance reveals spillover of SARS-CoV-2 genotypes into the community

**Figure 3:**
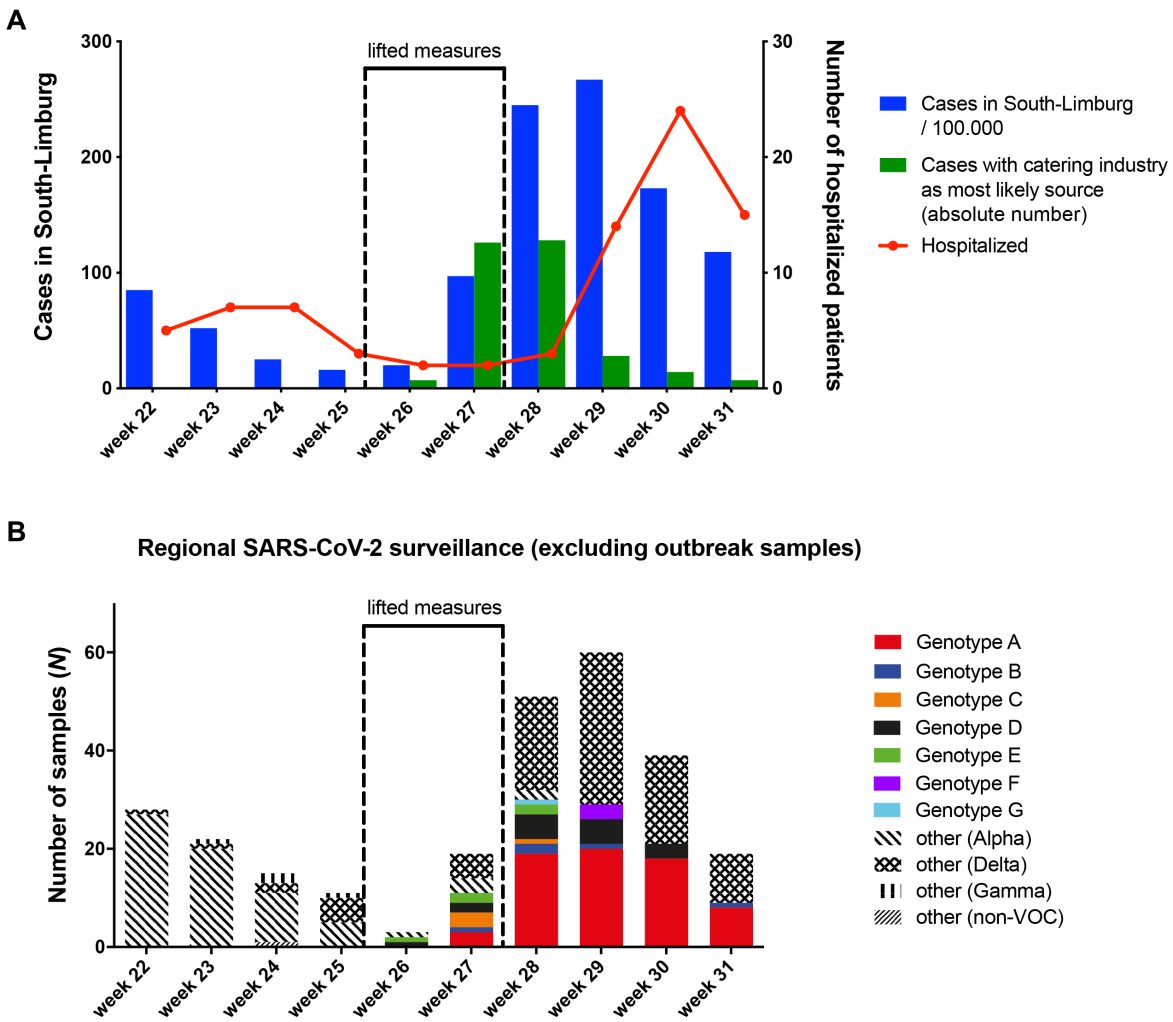
**(A)** Overview of SARS-CoV-2 incidence, cases linked to the bar and restaurant industry and weekly hospital admissions over time in South-Limburg. **(B)** Community circulation of SARS-CoV-2 genotypes that were associated with transmission clusters in the bar and restaurant industry based on genomic surveillance.

Community incidence of COVID-19 cases began to rise sharply in week 27, and dropped starting from week 30 after restrictions were reinstated at the end of week 27 (Figure 3A). The rise in community cases was associated with an increasing number of notified cases reporting a catering venue as the most likely source of their infection, peaking at n = 128 in week 28. The rise in community cases was followed by an increase of hospital admissions from 1.7/100,000 inhabitants to 50.2/100,000 inhabitants in week 30.

*Via* regional genomic surveillance based on random sampling, which is carried out to monitor the frequency of SARS-CoV-2 variants, it was possible to track the spread of the eight genotypes identified in the 16 venues into the community. Although genotype A was only established in 16% of surveillance samples in week 27, its frequency rose to over 30% in week 28 and 29, before peaking at 46% in week 30 (Figure 3B). Since this genotype was present in about 40% of outbreak samples in our study (all collected in week 26 and 27), these data suggest spillover of this genotype from the bar and restaurant industry into the community.

### Viral load in fully, partially, and non-vaccinated individuals

For 276 out of 348 (79%) cases in this study, the viral load was determined using the same laboratory workflow. No statistically significant difference in viral load between fully (median Ct value 16.41), partially (median Ct value 16.64) or non-vaccinated (median Ct value 15.44) cases was observed (Supplementary Figure S9).

## Discussion

Our analysis shows that lifting restrictions on bars and restaurants and the reopening of nightlife resulted in many cases linked to this setting. WGS revealed multiple clusters associated with specific venues and the proportion of cases that named the bar and restaurant industry as the most probable source of infection rose, suggesting that these were drivers of the increase in community incidence and increase in hospital admissions. The nightclub setting, high infectiousness of the Delta variant, high proportion of unvaccinated adults, the “Dansen met Janssen” campaign, many cases visiting multiple locations in a short time period, lack of compliance of the digital COVID-19 certificate, and lack of required testing for staff members, probably all contributed and should be addressed in order to safely open these venues when confronted with this issue in the future.

Nightclubs or nightclub/restaurant combinations had more linked cases than bars and restaurants, which can probably be explained by the closer and more frequent contacts and the higher mixing of contacts in a confined less ventilated space in nightclubs. A higher number of visitors could partly explain this difference, but WGS revealed multiple clusters linked to specific nightclubs pointing toward local transmission. The cluster comprising genotype E in venue 1 and venue 4 as well as the cluster with genotype A in venue 6 are clear examples with most cases in these locations comprising a similar genotype, suggesting these venues acted as locations for transmission. The combination of venue conditions on the one hand and the high infectiousness and shorter incubation period of the Delta variant probably contributed to rapid transmission in this setting.

This study shows that WGS is a powerful method for revealing transmission clusters and outbreaks, when used in combination with epidemiological investigations. Furthermore, thanks to continuous regional genomic surveillance of SARS-CoV-2, we were able to discriminate Delta variant genotypes associated with the outbreaks in the bar and restaurant industry from genotypes that were circulating in the community in the weeks prior to the opening of the bar and restaurant industry. Based on our results we conclude that lifting restrictions on the bar and restaurant industry was most likely responsible for the increase in community incidence and hospital admissions. In our study, the Delta variant and specifically genotype A, constituted a larger part of overall community incidence after the reopening of venues mirroring results from South-Korea, where many secondary cases were reported after the reopening of nightclubs (Kang et al., 2020). Additionally, the number of individuals that named a bar and restaurant industry venue as most probable source of their infection rose rapidly during our study period and quickly dropped after restrictions were reinstated. While none of our cases were hospitalized, hospital admissions rose 2 weeks after the increase in community incidence suggesting community transmission to vulnerable populations.

The policy that provided individuals with a digital corona entree ticket and the “Dansen met Janssen” campaign further contributed to the rapid spread as 17% of visitors that participate in this study did not require testing before entering a venue despite not being protected by their vaccination due to the short time elapsed after receiving their final vaccination dose (Table 2). The majority (47/53) had received the Janssen vaccine (Jcovden). During our study period, the Dutch government swiftly changed their policy to only giving a digital COVID-19 certificate 2 weeks after completing a vaccination scheme preventing similar situations to occur in the future.

Overall, 35% of visitor cases visited at least one venue during their contagious period, suggesting ineffectiveness of the digital COVID-19 certificate. Some venues like restaurants simply did not require the digital COVID-19 certificate for entering. Individuals who required testing 36 h prior to entering could still be in the incubation period of COVID-19 and therefore test negative and be contagious when entering the venue. Fully vaccinated individuals did not need to test prior to entry. Vaccination appears to reduce transmission of the Delta variant, but this reduction is less than in earlier variants (Cohn et al., 2021; CDC COVID-19 Vaccine Breakthrough Case Investigations Team, 2021; Lopez Bernal et al., 2021; Pouwels et al., 2021; Sheikh et al., 2021) and the impact of vaccination on transmission decreases over time (Levine-Tiefenbrun et al., 2021), meaning they could have contributed to transmission. No difference in viral load regardless of vaccination status and a higher proportion of asymptomatic infections among vaccinated individuals could have contributed with both of these results mirroring earlier studies (Acharya et al., 2022; Singanayagam et al., 2022). Because WGS analysis was not available for the majority of cases and not all visitors had been tested individual transmission pairs cannot be identified.

Lastly, other factors concerning the digital COVID-19 certificate contributed to rapid transmission. Thirty one percent of visitors visited multiple venues in one evening, increasing their chance of contracting or spreading COVID-19. Compliance of venues with the digital COVID-19 certificate was low. Multiple news reports mentioned falsified corona entree tickets or venues not checking the tickets. Unfortunately, it was not possible to analyze the proportion of cases that avoided testing prior to entering because no data on negative tests for the digital COVID-19 certificate exists.

Staff members were not required to have a digital COVID-19 certificate and probably contributed to overall transmission, as 60% (21/35) worked during their contagious period. Additionally, most affected venues had multiple staff members testing positive. Similarly, another study investigating a night club outbreak in Berlin determined a high attack rate among staff members (Muller et al., 2020), arguing for implementation of enhanced infection prevention and control measures, e.g., more stringent testing regimes for venue staff, exclusion of staff with symptoms.

Overall, our findings suggest that effectiveness of a digital COVID-19 certificate may be enhanced through more stringent control/authentication excluding falsified certificates, shortened validity of a negative (PCR) test result, and restricting visitors access to only one venue during a specified time period. For staff members, the use of a digital COVID-19 certificate is also recommended.

Our study has several strengths and limitations. One strength is the comprehensive analysis of all cases linked to venues in a specific location with a high number of samples being sequenced with many identified clusters. Secondly, the backdrop of our study of full reopening of nightlife in spite of a relatively high COVID-19 background incidence within a largely unvaccinated population offered a unique opportunity to investigate the role of these settings in transmission dynamics. Our first limitation is that we have no insight in overall attendance of these venues making the calculation of an attack rate impossible. Secondly, only a third of all cases were successfully sequenced meaning some transmission routes remained uncertain. Additionally, transmission routes outside of these venues were not analyzed. Thirdly, venues with <10 cases were not investigated thereby excluding the potential role that smaller bars and restaurants may have had in the outbreak. Lastly, data on the reported setting where infection most probably occurred is self-reported, meaning that the true proportion of cases infected at venues could be different.

By combining epidemiological and WGS data, we were able to reveal multiple transmission clusters in the bar and restaurant industry and identified venues with dancing facilities as the drivers of SARS-CoV-2 transmission. Lifting restrictions on bar and restaurant industry venues with a corona entree ticket in a population where less than 20% was fully vaccinated led to a surge in COVID-19 cases and promoted the spread of new variants. In the future, careful consideration is necessary when opening settings that pose a high risk for transmission during periods of low vaccination coverage and high population prevalence.

## Data availability statement

The datasets presented in this study can be found in online repositories. The names of the repository/repositories and accession number(s) can be found at: ENA - PRJEB61626.

## Author contributions

BV, KG, CaH, VH, LA, PS, ChH, and JD contributed to the conceptualization and the design of the study. BV, KG, and JD drafted the manuscript. BV and JD performed data analysis. KG interviewed the venues and individuals. BV and JD contributed to the visualization of the results. JD coordinated the research done in this manuscript. Interpretation and critical revision of the manuscript was done by BV, KG, CaH, VH, LA, PS, ChH, and JD. BV, KG, CaH, VH, LA, PS, ChH, and JD had access to the analyzed datasets, provided approval to submit the manuscript for publication, and also had access to verify the raw data. All authors contributed to the article and approved the submitted version.

## Conflict of interest

The authors declare that the research was conducted in the absence of any commercial or financial relationships that could be construed as a potential conflict of interest.

## Publisher’s note

All claims expressed in this article are solely those of the authors and do not necessarily represent those of their affiliated organizations, or those of the publisher, the editors and the reviewers. Any product that may be evaluated in this article, or claim that may be made by its manufacturer, is not guaranteed or endorsed by the publisher.

## Supplementary material

The Supplementary material for this article can be found online at: https://www.frontiersin.org/articles/10.3389/fmicb.2023.1183877/full#supplementary-material
