# Supplementary material for: SARS-CoV-2 transmission dynamics in bars, restaurants, and nightclubs
Source: Front Microbiol. 2023 May 18;14:1183877. doi: 10.3389/fmicb.2023.1183877 (PMC10232797; doi:10.3389/fmicb.2023.1183877)
Supplement: Supplementary file 6 [file Data_Sheet_1.PDF]

Supplementary Material

SARS-CoV-2 transmission dynamics in bars, restaurants and nightclubs

This PDF file includes:

Supplemental Figures S1 to S9
Table S1
Captions for Movie S1
Captions for Data S1 to S4

Other Supplementary Materials for this manuscript include the following:

Movie S1
Data S1 to S4

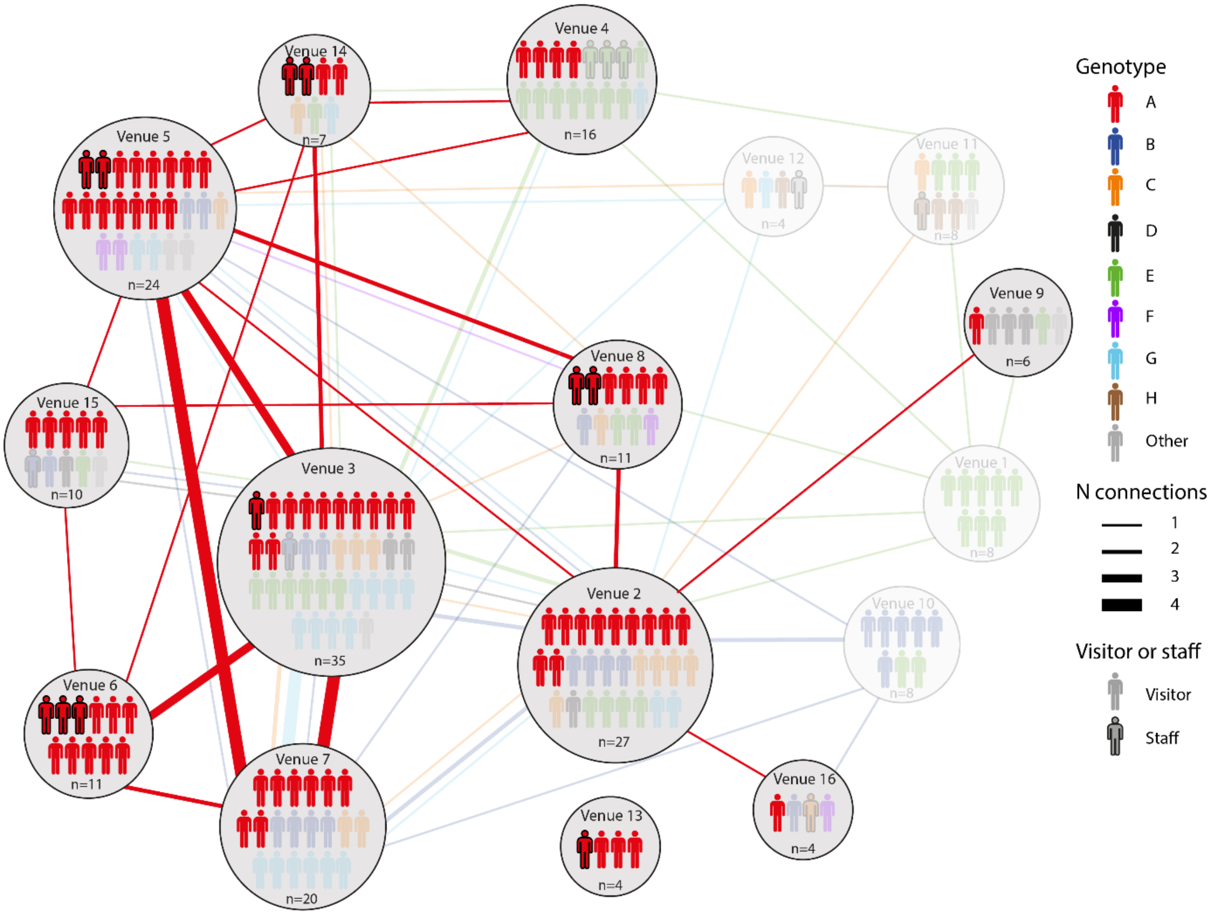

Figure S1. Overview of the venues that included cases that were infected with genotype A. Lines between venues indicate that a particular case visited both venues.

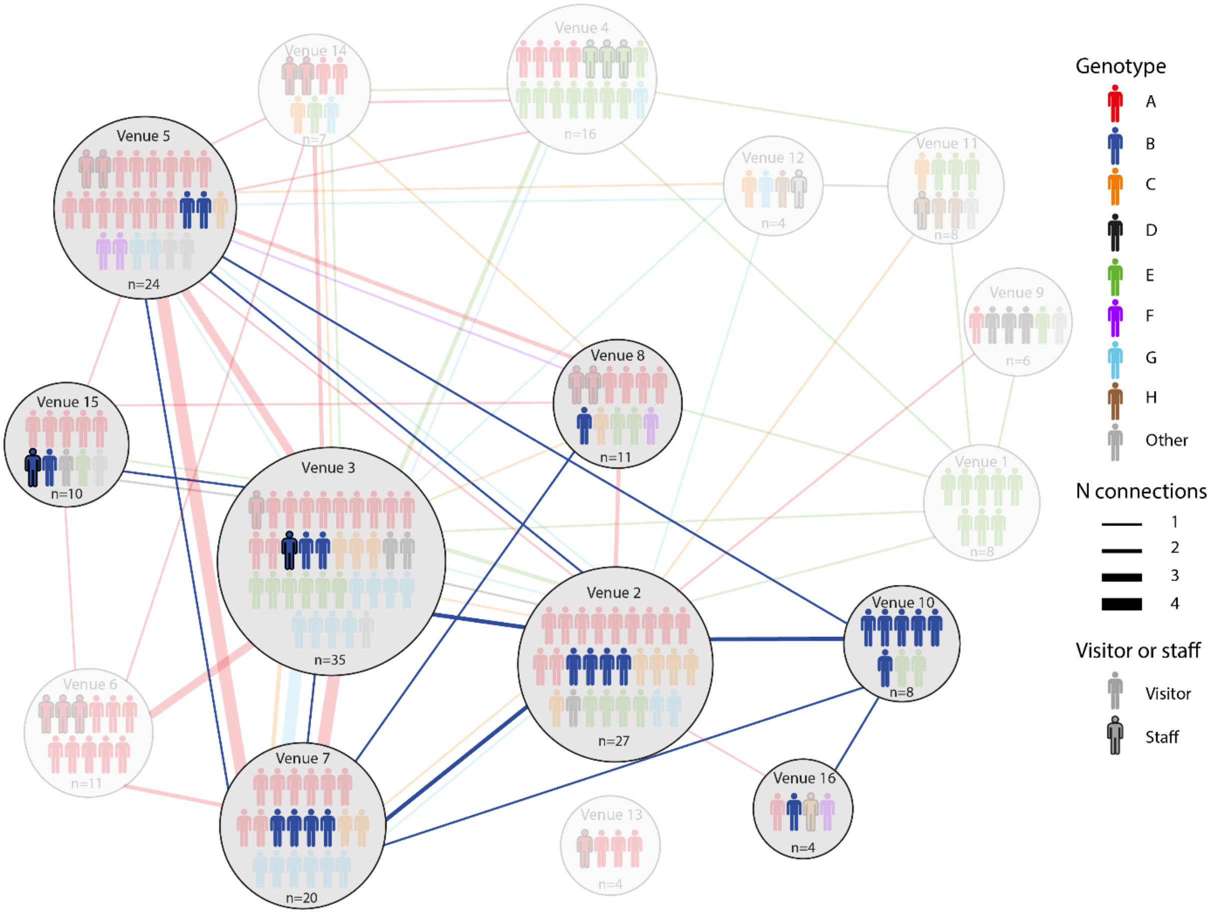

Figure S2. Overview of the venues that included cases that were infected with genotype B. Lines between venues indicate that a particular case visited both venues.

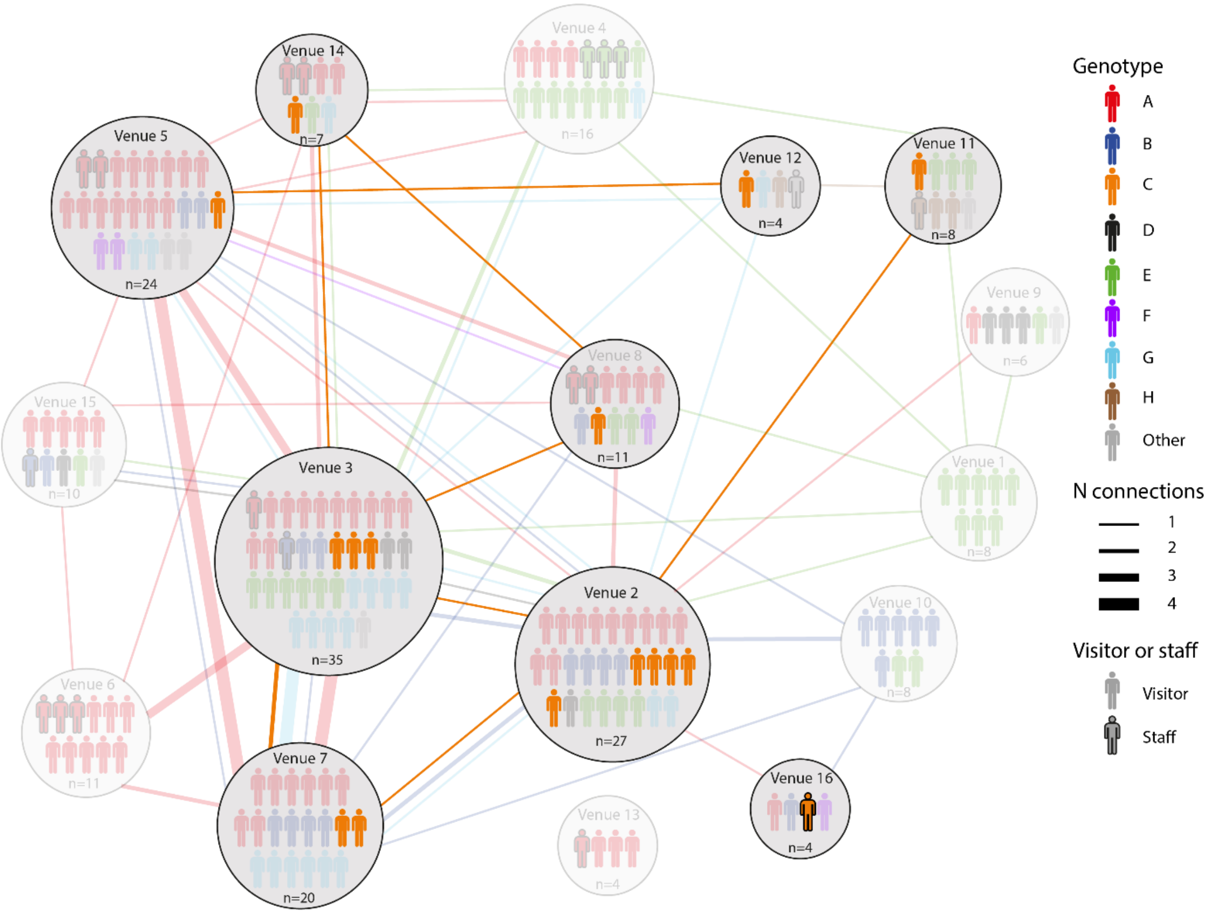

Figure S3. Overview of the venues that included cases that were infected with genotype C. Lines between venues indicate that a particular case visited both venues.

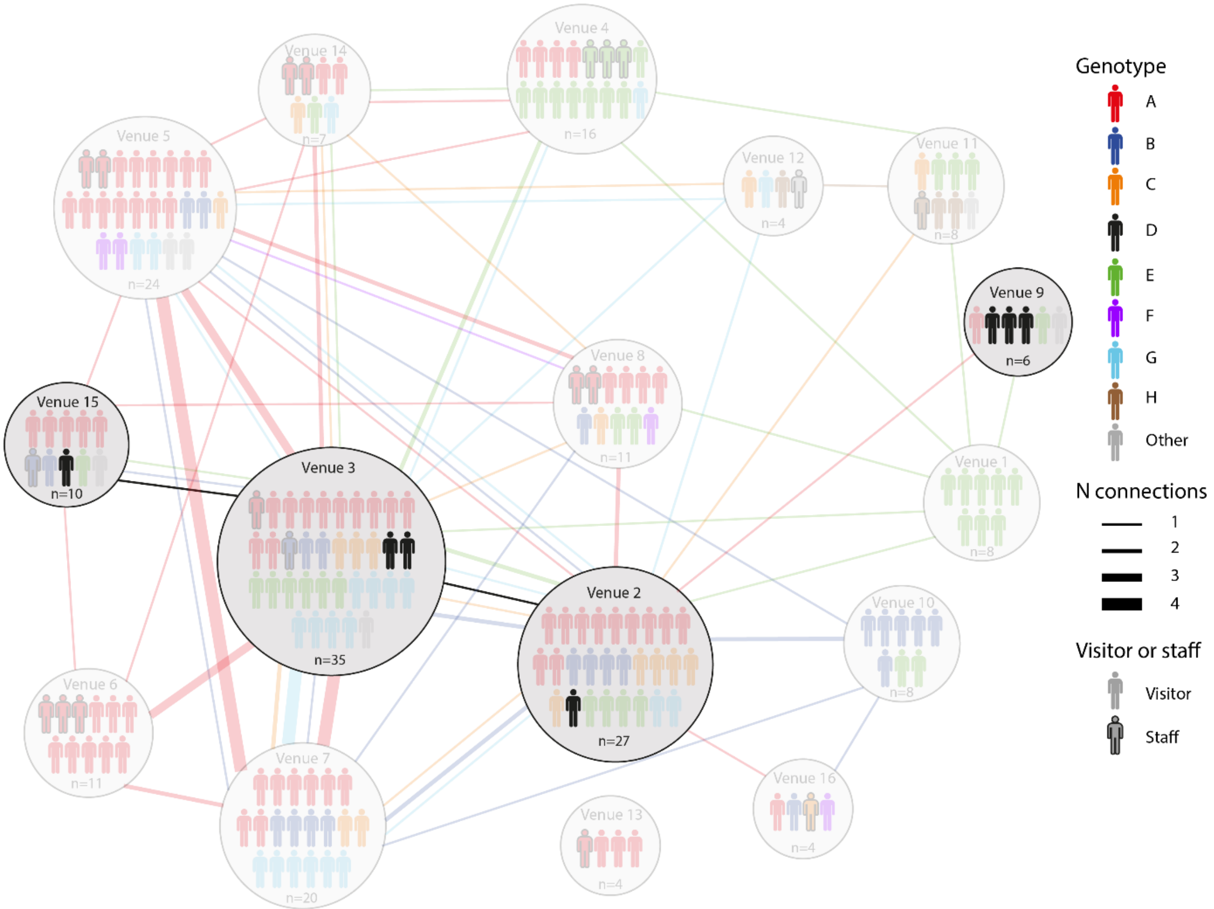

Figure S4. Overview of the venues that included cases that were infected with genotype D. Lines between venues indicate that a particular case visited both venues.

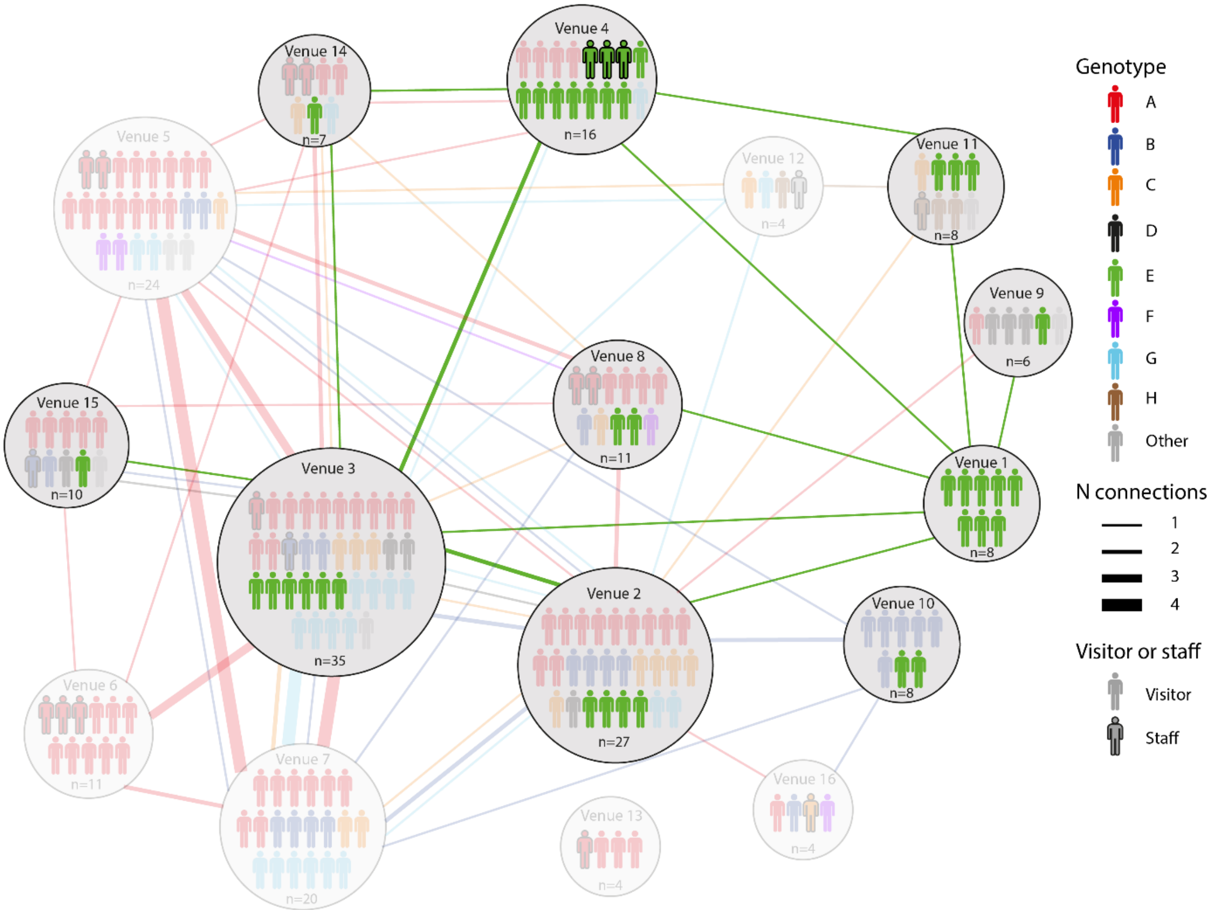

Figure S5. Overview of the venues that included cases that were infected with genotype E. Lines between venues indicate that a particular case visited both venues.

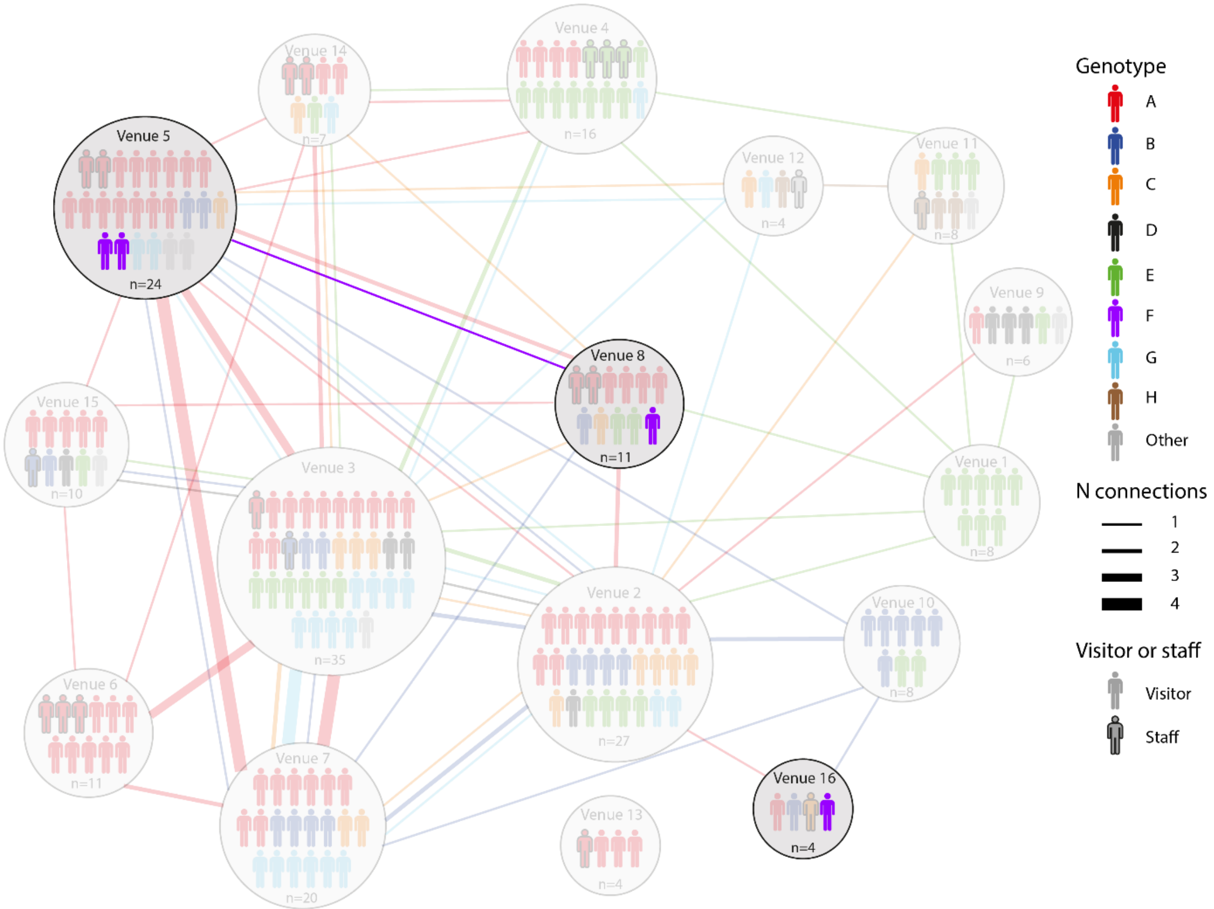

Figure S6. Overview of the venues that included cases that were infected with genotype F. Lines between venues indicate that a particular case visited both venues.

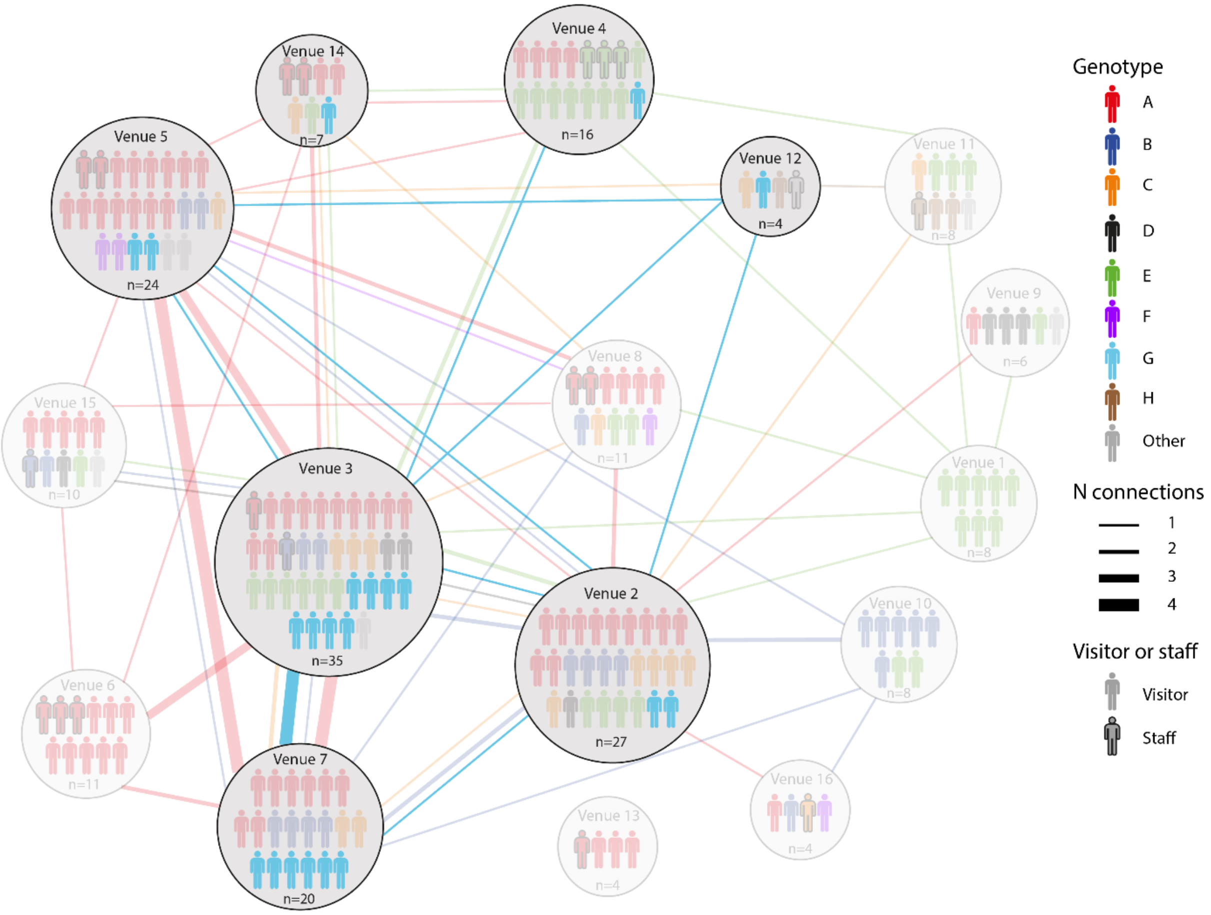

Figure S7. Overview of the venues that included cases that were infected with genotype G. Lines between venues indicate that a particular case visited both venues.

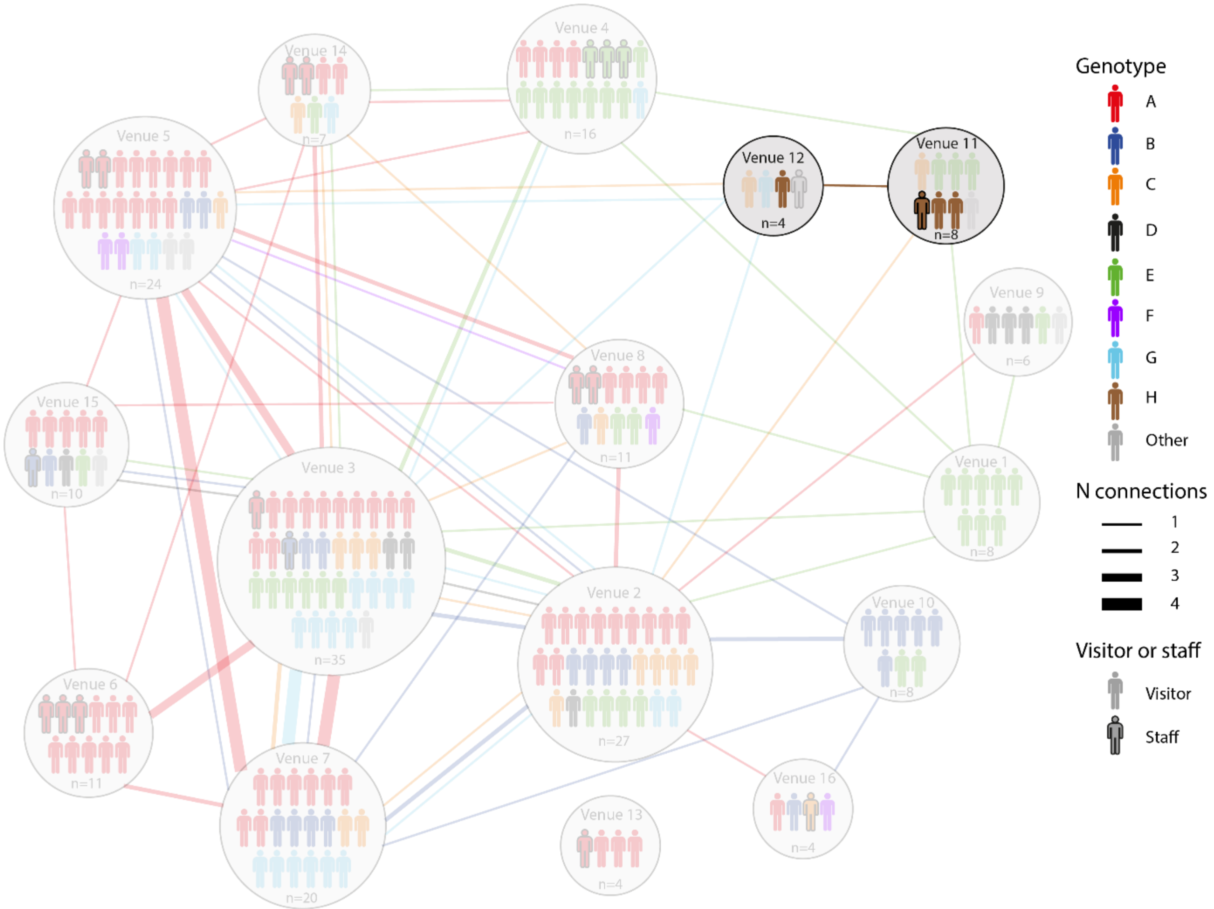

Figure S8. Overview of the venues that included cases that were infected with genotype H. Lines between venues indicate that a particular case visited both venues.

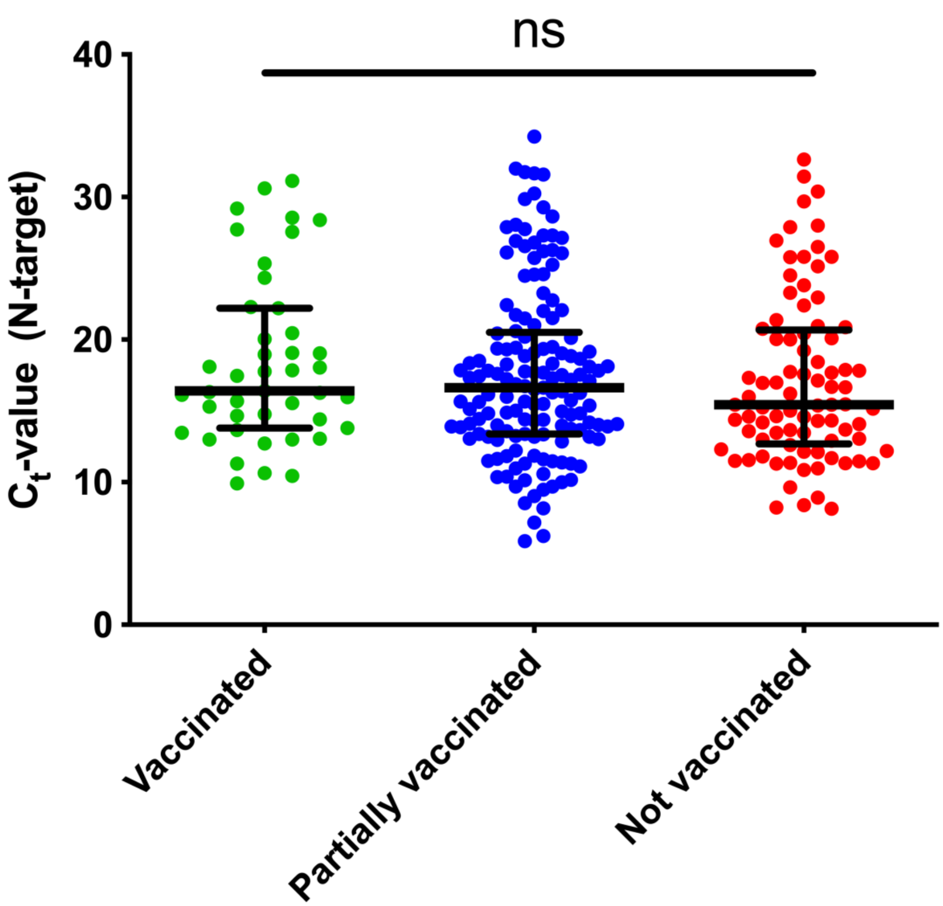

Figure S9. Viral load (expressed as cycle threshold values) of vaccinated, partially vaccinated and non-vaccinated cases.

Table S1. Overview of linked cases and number of links with other venues per venue.

Venue	# of linked cases (same night)	# of venues with link (same night)	# of venues with link (total)	Total cases
**1**	6	6	6	46
**2**	18	9	10	63
**3**	29	9	10	98
**4**	6	4	5	44
**5**	15	6	9	61
**6**	4	3	4	32
**7**	15	5	6	50
**8**	8	5	6	42
**9**	2	2	2	11
**10**	1	1	4	18
**11**	3	3	4	15
**12**	4	3	4	10
**13**	0	0	0	14
**14**	6	4	5	15
**15**	4	2	4	27
**16**	0	0	2	14

Movie S1. Time-lapse movie of the movement of each successfully genotyped case between venues.

Data S1. (separate file)
Source data Figure 1, Figures S1-S9, Table S1 & Movie S1

Data S2. (separate file)
Source data Figure 2A

Data S3. (separate file)
Source data Figure 2B

Data S4. (separate file)
Source data Figure 3
